# Use of clinical variables for preoperative prediction of lymph node metastasis in endometrial cancer

**DOI:** 10.1093/jjco/hyad135

**Published:** 2023-10-09

**Authors:** Yuta Ueno, Emiko Yoshida, Shuko Nojiri, Tomoyasu Kato, Takashi Ohtsu, Toshiyuki Takeshita, Shunji Suzuki, Hiroshi Yoshida, Ken Kato, Masayoshi Itoh, Tsuguto Notomi, Kengo Usui, Takashi Sozu, Yasuhisa Terao, Hideya Kawaji, Hisamori Kato

**Affiliations:** Department of Gynecology, Kanagawa Cancer Center, Yokohama, Japan; Department of Obstetrics and Gynecology, Nippon Medical School, Tokyo, Japan; Department of Obstetrics and Gynecology, NTT Medical Center Tokyo, Tokyo, Japan; Department of Obstetrics and Gynecology, Juntendo University, Tokyo, Japan; Medical Technology Innovation Center, Juntendo University, Tokyo, Japan; Clinical Research and Trial Center, Juntendo University, Tokyo, Japan; Department of Gynecology, National Cancer Center, Tokyo, Japan; Division of Advanced Cancer Therapeutics, Kanagawa Cancer Center Research Institute, Yokohama, Japan; Center for Cancer Genome Medicine, Kanagawa Cancer Center, Yokohama, Japan; Department of Obstetrics and Gynecology, Nippon Medical School, Tokyo, Japan; Department of Obstetrics and Gynecology, Nippon Medical School, Tokyo, Japan; Department of Diagnostic Pathology, National Cancer Center, Tokyo, Japan; Clinical Research Support Office, Biobank Translational Research Support Section, National Cancer Center Hospital, Tokyo, Japan; Laboratory for Advanced Genomics Circuit, RIKEN Center for Integrative Medical Sciences, Yokohama, Japan; Department of Obstetrics and Gynecology, NTT Medical Center Tokyo, Tokyo, Japan; Medical Technology Innovation Center, Juntendo University, Tokyo, Japan; Department of Information and Computer Technology, Faculty of Engineering, Tokyo University of Science, Tokyo, Japan; Department of Obstetrics and Gynecology, Juntendo University, Tokyo, Japan; Research Center for Genome and Medical Sciences, Tokyo Metropolitan Institute of Medical Science, Tokyo, Japan; Department of Gynecology, Kanagawa Cancer Center, Yokohama, Japan; Kanagawa Health Service Association, Yokohama, Japan

**Keywords:** endometrial cancer, lymphadenectomy, lymph node metastasis, probabilistic prediction, risk factor

## Abstract

**Objective:**

Endometrial cancer is the most common gynaecological cancer, and most patients are identified during early disease stages. Noninvasive evaluation of lymph node metastasis likely will improve the quality of clinical treatment, for example, by omitting unnecessary lymphadenectomy.

**Methods:**

The study population comprised 611 patients with endometrial cancer who underwent lymphadenectomy at four types of institutions, comprising seven hospitals in total. We systematically assessed the association of 18 preoperative clinical variables with postoperative lymph node metastasis. We then constructed statistical models for preoperative lymph node metastasis prediction and assessed their performance with a previously proposed system, in which the score was determined by counting the number of high-risk variables among the four predefined ones.

**Results:**

Of the preoperative 18 variables evaluated, 10 were significantly associated with postoperative lymph node metastasis. A logistic regression model achieved an area under the curve of 0.85 in predicting lymph node metastasis; this value is significantly higher than that from the previous system (area under the curve, 0.74). When we set the false-negative rate to ~1%, the new predictive model increased the rate of true negatives to 21%, compared with 6.8% from the previous one. We also provide a spreadsheet-based tool for further evaluation of its ability to predict lymph node metastasis in endometrial cancer.

**Conclusions:**

Our new lymph node metastasis prediction method, which was based solely on preoperative clinical variables, performed significantly better than the previous method. Although additional evaluation is necessary for its clinical use, our noninvasive system may help improve the clinical treatment of endometrial cancer, complementing minimally invasive sentinel lymph node biopsy.

## Teaser

A probabilistic predictive model of lymph node metastasis in endometrial cancer based on preoperative clinical variables was developed. It has the potential to enhance clinical treatment by reducing unnecessary lymphadenectomy.

## Introduction

Accounting for >417 000 new cases and 97 000 deaths worldwide in 2020, endometrial cancer is the fourth most common cancer diagnosed in women and the most common gynaecologic cancer in developed countries (1). The incidence of endometrial cancer has risen globally over the past 30 years owing to changes in lifestyle, socioeconomic factors and the growing epidemic of obesity (2). In Japan, the number of new cases of endometrial cancer during the 1970s was ~1000 annually and increased to >5000 in 1999 and 10 000 in 2015. In 2007, morbidity due to endometrial cancer exceeded that of cervical cancer, making endometrial cancer the most common gynaecologic cancer (3,4). Currently, most cases of endometrial cancer are identified while the disease is still in its early stages, and the mortality rate associated with endometrial cancer is the lowest among gynaecologic cancers (5).

Surgery is a key component of the diagnosis of endometrial cancer. In particular, staging based on surgical findings has been a critical step in the clinical management of endometrial cancer since 1988, when the International Federation of Gynecology and Obstetrics (FIGO) system was introduced. According to FIGO2008—the current diagnostic system—74.4% of cases of endometrial cancer in Japan in 2020 were stage I, 5.1% were stage II, 12.0% were stage III and 8.4% were stage IV (5). In addition, the main adverse prognostic factor for endometrial cancer is lymph node metastasis (LNM), which is evaluated surgically through conventional lymph node biopsy, sentinel lymph node (SLN) biopsy and systemic lymphadenectomy. Any type of lymphadenectomy is associated with a risk of lymphedema, which poses significant challenges for endometrial cancer patients. The risk of lower extremity lymphedema after lymphadenectomy for endometrial cancer ranges from 10 to 20%, and the risk of lymphocele development ranges from 10 to 25% (6–11).

In addition to its roles in diagnosis and staging, surgery is a component of the standard treatment protocol for endometrial cancer, which includes total hysterectomy and bilateral adnexectomy. To reduce the risk of metastasis, some patients warrant systemic lymphadenectomy, such as pelvic or paraaortic lymphadenectomy. In previous studies, the survival of high-risk patients was correlated with the number of dissected lymph nodes (12) and improved when pelvic lymphadenectomy was combined with removal of the paraaortic nodes (13). However, low-risk patients may receive little therapeutic benefit from lymphadenectomy (14,15). Even though the therapeutic benefit of lymphadenectomy for endometrial cancer is under debate (12,14,15), the diagnosis of LNM—which requires lymphadenectomy—remains important in establishing prognosis. A risk-based approach could inform the appropriate extent of systemic lymphadenectomy in individual patients (16).

Therefore, noninvasive preoperative staging and LNM assessment are anticipated to improve the quality of clinical treatment for endometrial cancer. Several recent studies have used only clinical factors, such as myometrial invasion and tumour pathology, to indirectly assess LNM without lymphadenectomy. These studies have led to the development of several scoring systems (17–21). In particular, the Kanagawa Cancer Center (KCC) system was proposed as a means for directing the extent of lymphadenectomy in patients with endometrial cancer (17,22–24). The system considers the presence of four factors associated with metastasis: histological type (other than endometrial carcinoma G1), tumour volume (>6 cm^3^), serum carbohydrate antigen 125 (CA125) level (premenopausal, ≥70 U/ml; postmenopausal, ≥25 U/ml) and myometrial invasion (<1/2 of the thickness of the myometrium, or more). Each factor is assigned 1 point, and the KCC score ranges from 0 to 4 points. The extent of lymphadenectomy is guided according to the resulting score: no lymphadenectomy for patients with 0 points, pelvic lymphadenectomy for patients with scores of 1 or 2 points and pelvic lymphadenectomy with para-aortic lymphadenectomy for those with 3 or 4 points.

Although these previous scoring systems were constructed to be simple enough to use in clinical practice, they have not been validated through multi-institutional studies. Moreover, several questions remain, including: Are any other clinical variables useful in addition to the four mentioned earlier? and Is any improvement in LNM prediction accuracy achieved by using a statistical model instead of simple point counting? Here, we report the findings of a multi-institutional study that systematically examined 18 preoperative clinical variables associated with LNM. We used the relevant variables to construct a statistical model for predicting LNM in endometrial cancer and explored its potential in sample cases. We also provide a tool for calculating the LNM risk score in forthcoming patients with endometrial cancer.

## Patients and methods

### Ethics approval and consent to participate

The design of this study was reviewed and approved by the Ethics Committee for Clinical Studies of the Juntendo University of Medicine (M18-0093), which covered Juntendo University (the main facility), Juntendo University Shizuoka, Juntendo University Urayasu and Juntendo University Nerima. Additional ethics approval was obtained at each of the participating hospitals and research institutes [KCC, National Cancer Center Japan (NCC), RIKEN, Tokyo University of Science, Nippon Medical School and Tokyo Metropolitan Institute of Medical Science]. All procedures in studies involving human participants were performed in accordance with the ethical standards of each institutional research committee and with the 1964 Declaration of Helsinki and its subsequent amendments or comparable ethical standards. Written informed consent was obtained from all patients.

### Patients

The study population was drawn from the 765 patients with endometrial cancer who had undergone initial surgery at the hospitals of KCC, NCC, Juntendo University, Juntendo University Shizuoka, Juntendo University Urayasu, Juntendo University Nerima or Nippon Medical School between 2006 and 2019. These seven hospitals were allocated to four groups according to various characteristics: university hospitals (UNI) that provide multidisciplinary and advanced cancer treatment and serve as the university’s main hospital (Juntendo University and Nippon Medical School); municipal hospitals (MNI) that provide community-based cancer treatment as branches of Juntendo University (Juntendo University Shizuoka, Juntendo University Urayasu and Juntendo University Nerima); KCC and NCC.

Data regarding the following 18 preoperative variables were collected for each patient: demographic information [age, body mass index (BMI), gravidity, parity, menopause and history of breast cancer and colorectal cancer], serologic markers [CA125, carbohydrate antigen 19-9 (CA19-9) and carcinoembryonic antigen (CEA)], imaging-based findings (tumour volume index, myometrial invasion, cervical stromal invasion, adnexal involvement, enlarged pelvic or para-aortic lymph nodes and distal metastasis) and FIGO2008-based pathologic diagnosis (classified as endometrial carcinoma other than G1). The cases with enlarged pelvic and para-aortic lymph nodes were separately handled as a previous study implied potential difference of risk levels (17). Two levels classification of pathology (G1 or the others) were employed for the concordance with the previous studies (17,22–24). Imaging studies followed clinical practice at the individual hospitals, and the collected information came from either magnetic resonance imaging (MRI), computed tomography (CT) or positron emission tomography (PET). Tumour volume index was defined as the product of the maximum diameter along the uterine axis in a sagittal section image, the maximum anteroposterior diameter in an axial section image and the maximum horizontal diameter in an axial section image. In addition, data on the following postoperative features were collected: myometrial invasion, cervical stromal invasion, adnexal involvement, histologic type, other pathologic findings and LNM. Data collection of the patients recruited in NCC is supported by the National Cancer Center Biobank, Japan.

### Statistical analysis

All analyses were conducted by using R version 4.1.2. Continuous variables of the patient characteristics were indicated as mean (standard deviation) in Table 1 and compared among four institution groups by using one-way analysis of variance. Categorical variables of the patient characteristics were expressed as number of cases (% proportion) and compared by using Fisher’s exact test. Logistic regression (LR) models were used to predict the postoperative LNM, where continuous variables except for age were logarithmically transformed with base 2 considering their interpretation of odd ratio derived from the LR models. Variables included in the multivariate LR model were selected based on their association with and predictive performance of the postoperative LNM, and manual curation of their clinical relevance. Random forest modelling was conducted by using randomForest package (default settings). As a measure of predictive performance, area under the curve (AUC) values were calculated using pROC package, and their 95% confidence intervals (CIs) were calculated using bootstrap method with 2000 replicates (default settings).

**Table 1:** Collected clinical variables

Clinical variables	KCC	MNI	NCC	UNI	
	(*n* = 199)	(*n* = 46)	(*n* = 196)	(*n* = 170)	*P* value
Demographic characteristic					
Age (years old)	60.1 (9.5)	56.7 (10.4)	58.3 (10.5)	57.1 (9.6)	0.017
BMI (kg/m^2^)	23.5 (4.7)	23.9 (4.5)	23.9 (4.9)	24.2 (5.8)	0.567
Gravidity	149 (74.9%)	31 (67.4%)	135 (68.9%)	106 (62.4%)	0.078
Parity	142 (71.4%)	28 (60.9%)	127 (64.8%)	96 (56.5%)	0.027
Menopause	156 (78.4%)	33 (71.7%)	137 (69.9%)	113 (66.5%)	0.067
History of breast cancer	7 (3.5%)	2 (4.3%)	16 (8.2%)	9 (5.3%)	0.256
History of colorectal cancer	0 (0.0%)	1 (2.2%)	8 (4.1%)	1 (0.6%)	0.004
Serologic markers					
CA125 (U/ml)	93.5 (363.9)	48.2 (65.9)	52.5 (81.9)	55.5 (129.7)	0.225
CA19-9 (U/ml)	96.9 (458.8)	54.0 (83.8)	205.3 (1089.0)	89.8 (326.8)	0.276
CEA (U/ml)	2.8 (3.3)	5.0 (12.2)	3.1 (7.4)	3.0 (6.5)	0.269
Imaging-based findings					
Tumour volume index (cm^3^)	62.9 (99.1)	73.0 (101.0)	73.6 (118.5)	38.5 (57.4)	0.004
Myometrial invasion	107 (53.8%)	15 (32.6%)	79 (40.3%)	69 (40.6%)	0.008
Cervical stromal invasion	4 (2.0%)	2 (4.3%)	28 (14.3%)	10 (5.9%)	<0.001
Adnexal involvement	4 (2.0%)	1 (2.2%)	8 (4.1%)	5 (2.9%)	0.696
Enlarged pelvic LN	4 (2.0%)	8 (17.4%)	25 (12.8%)	32 (18.8%)	<0.001
Enlarged paraaortal LN	7 (3.5%)	1 (2.2%)	11 (5.6%)	13 (7.6%)	0.288
Distal metastasis	0 (0.0%)	1 (2.2%)	5 (2.6%)	4 (2.4%)	0.081
Pathological preoperative diagnosis					
Pathology, non-G1	101 (50.8%)	19 (41.3%)	77 (39.3%)	63 (37.1%)	0.038
Postoperative diagnosis					
LNM	34 (17.1%)	5 (10.9%)	36 (18.4%)	29 (17.1%)	0.716
LNM type					
Pelvic LN	15 (7.5%)	2 (4.3%)	20 (10.2%)	20 (11.8%)	
Paraaortal LN	5 (2.5%)	1 (2.2%)	3 (1.5%)	1 (0.6%)	
Pelvic and paraaortal LN	14 (7.0%)	2 (4.3%)	13 (6.6%)	8 (4.7%)	
Myometrial invasion	85 (42.7%)	14 (30.4%)	91 (46.4%)	69 (40.6%)	0.239
Cervical stromal invasion	25 (12.6%)	5 (10.9%)	41 (20.9%)	19 (11.2%)	0.039
Adnexa involvement	15 (7.5%)	1 (2.2%)	21 (10.7%)	10 (5.9%)	0.178
Pathology, non-G1	123 (61.8%)	30 (65.2%)	94 (48.0%)	74 (43.5%)	0.001

Data are shown as no. (%) of cases for categorical variables and as mean (standard deviation) for continuous variables.

## Results

### Clinicohistologic characteristics

**Figure 1:**
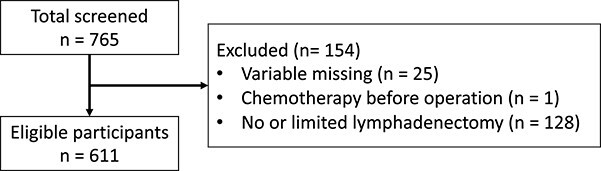
Recruited patients.

We recruited 765 patients with endometrial cancer from seven hospitals, comprising the following four groups: KCC, NCC, UNI and MNI (see Methods for details). Of these, 611 eligible patients remained after we excluded 154 cases because of missing variables (25 cases), preoperative chemotherapy (one case) and surgery with no or limited lymphadenectomy (129 cases) (Fig. 1). For each case, we collected information on the 18 preoperative variables comprising seven demographic variables, three serologic markers, seven imaging-based observations and pathologic diagnosis (i.e. endometrial carcinoma other than G1, according to the FIGO2008 classification). We also collected data regarding five diagnostic results including LNM as postoperative variables (see Methods). We noted several differences in these data across study sites and disease stages.

### Univariate analysis identified preoperative variables associated with LNM in endometrial cancer

We first evaluated the statistical association of the 18 preoperative variables with LNM as a postoperative (definitive) diagnosis. Univariate LR analysis defined 10 variables as significantly associated with LNM (Table 2). As expected, the four variables used in the KCC scoring system (KCC score)—i.e. pathologic diagnosis, myometrial invasion, tumour volume index and CA125 level—were included among the 10 significant variables. Other significant variables included cervical stromal invasion and the three imaging-based findings beyond the uterine corpus—enlarged pelvic lymph node, enlarged paraaortic lymph node and distal metastasis. However, the remaining imaging-based variable that we evaluated, adnexal involvement, was not significantly associated with LNM. Like LNM, adnexal involvement is a feature of FIGO stage 3 disease, and its association with LNM is pathologically expected. In addition, the few cases of preoperative diagnosis of adnexal involvement in our study population (3.2% of patients; 20 cases) may likewise indicate the difficulty of diagnosing this disease feature and its unlikely usefulness as a prognostic factor for LNM. Our cohort also showed significant associations of BMI and CA19-9 level with LNM in endometrial cancer, as reported in other studies (25,26). However, although a previous study suggested the association of CEA levels with LNM (27), neither our current study nor other follow-up studies support this earlier finding.

**Table 2:** Univariate logistic regression analysis of preoperative variables

Preoperative variables	Odds ratio	95% CI^*^ (lower)	95% CI^*^ (higher)	*P* value
Age	1.00	0.98	1.02	0.877
BMI (log_2_)	2.58	1.27	5.27	0.009
Gravidity	0.78	0.50	1.23	0.279
Parity	0.82	0.54	1.28	0.382
Menopause	0.86	0.55	1.37	0.515
History of breast cancer	0.29	0.05	0.98	0.094
History of colorectal cancer	1.22	0.18	4.97	0.801
CA125 (U/ml, log_2_)^a^	1.75	1.53	2.03	<0.001
CA19-9 (U/ml, log_2_)	1.17	1.07	1.28	<0.001
CEA (U/ml, log_2_)	1.19	0.99	1.41	0.053
Tumour volume index (cm^3^, log_2_)^a^	1.57	1.38	1.80	<0.001
Myometrial invasion^a^	3.51	2.25	5.58	<0.001
Cervical stromal invasion	2.78	1.40	5.32	0.003
Adnexal involvement	1.41	0.39	4.02	0.553
Enlarged pelvic LN	6.55	3.84	11.21	<0.001
Enlarged paraaortal LN	18.71	8.46	45.82	<0.001
Distal metastasis	21.04	5.18	140.85	<0.001
Pathology, non-G1^a^	2.09	1.37	3.22	0.001

*CI*, confidence interval

^a^Used in KCC score.

### Performance of individual variables in predicting LNM in endometrial cancer

**Figure 2:**
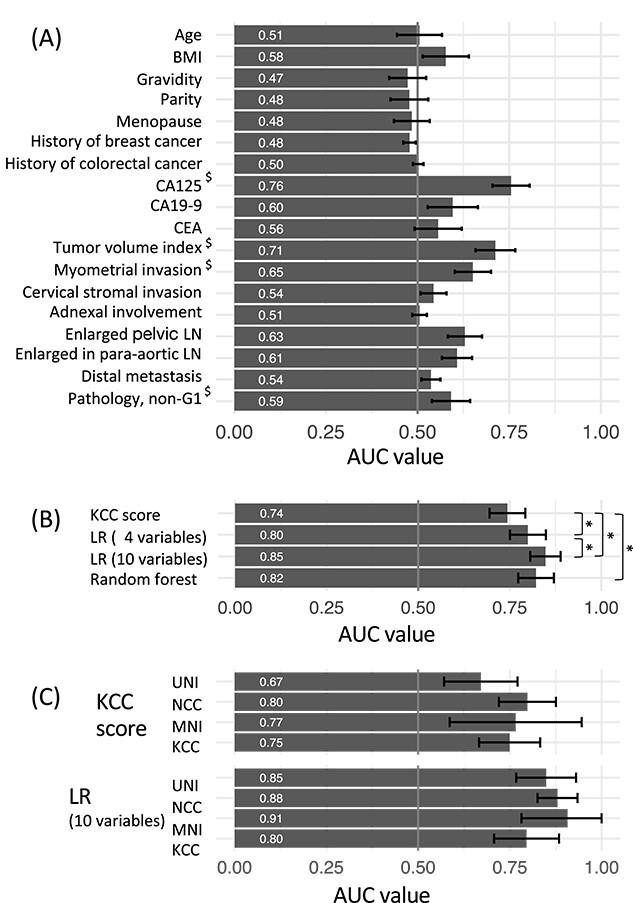
Performance of predictive models. (A) Area under the curve (AUC value) of single-variable predictive models. Error bars, 95% confidence intervals; $, variable used in the KCC scoring system. (B) AUC values of predictive models with multiple variables. Logistic regression (LR) models used the same four variables as in the KCC scoring method or the 10 significant clinical variables identified in the current study; the random forest model included the same 10 clinical variables. *Significant (*P* < 0.05) difference between the two predictive models after bootstrapping; CA, carbohydrate antigen; CEA, carcinoembryonic antigen; LN, lymph node. (C) AUC values according to study site. KCC, Kanagawa Cancer Center; MNI, municipal hospitals; NCC, Japan’s National Cancer Center; UNI, university hospitals.

We next evaluated the performance of individual variables as prognostic factors for LNM according to the AUC values obtained through receiver operating characteristic analysis (Fig. 2A). The three variables with the highest predictive performance were CA125 (AUC, 0.76), tumour volume index (AUC, 0.71) and myometrial invasion (AUC, 0.65). All of these variables are included in the KCC scoring system, and our current result from a multi-institutional cohort provides independent evidence supporting the relevance of the KCC score. In addition, the AUC values for CA19-9 (AUC, 0.60) and the presence of enlarged pelvic and para-aortic lymph nodes (AUC, 0.63 and 0.61, respectively) were higher than that for the remaining KCC score variable, specifically pathologic diagnoses other than G1 (AUC, 0.59). These factors are often assessed in clinical practice, consistent with their utility in prognostic prediction. Similarly, the inclusion of cervical invasion (AUC, 0.54) and distal metastasis (AUC, 0.54) in a prognostic prediction scheme is plausible in light of their frequent clinical use, even though their AUCs for were slightly lower than that for pathologic diagnosis. Notably, the performance of BMI (AUC, 0.58) is very close to that for the KCC variable of pathologic diagnosis. Although a modest change in BMI likely would not influence LNM risk, extreme cases of overweight and obesity have been associated with this outcome (25). Therefore, BMI is likely useful as a prognostic factor of LNM in endometrial cancer.

### Multivariate statistical models outperformed the KCC scoring system

To facilitate accurate prediction of LNM in endometrial cancer according to preoperative variables, we constructed two multivariate LR models. One was based on the 10 variables revealed as useful in our univariate assessment, and the other used the four variables in the KCC score. In addition, we used the 10 significantly associated preoperative variables to construct a nonlinear model by using randomForest analysis (28), a recently developed machine learning algorithm.

Regarding the LNM predictive performance of our three statistical models (Fig. 2B), the KCC score achieved an AUC of 0.74 (95% CI, 0.69–0.79), which exceeded that of most single-variable models (Fig. 2A) and indicated the benefit of combining variables. Our LR model using the same four variables as in the KCC system achieved an AUC of 0.80 (95% CI, 0.75–0.85), a significant improvement (*P* = 6.1e-05) over the KCC score. Furthermore, the LR model using all 10 variables achieved an AUC of 0.85 (95% CI, 0.81–0.89), which was significantly (*P* = 0.001) greater than that of the four-variable model and suggests the substantial contributions of the additional six variables. Overall, the performance of the 10-variable regression model was highly significantly (*P* = 1.3e-07) improved over the KCC score. However, a randomForest model with the same 10 variables (AUC, 0.82; 95% CI, 0.77–0.87) showed similar predictive performance to the LR model, suggesting that an AUC of 0.84 was likely the upper limit for our current dataset, regardless of the model applied.

Because our cohort consisted of patients in four institutional groups, we further examined the performance of the 10-variable LR model compared with the KCC score across study sites (Fig. 2C). The results from both predictive models varied somewhat depending on the cohort subset, thus indicating the influence of various patient characteristics. Notably, the LR model generally achieved higher performance and smaller variance than the KCC score. This pattern likely reflects the contributions of the additional variables and the effect of continuous values on the resulting score (probability) from the LR model, whereas the KCC scores were discrete values from 0 to 4.

### Increased ‘metastasis-free’ diagnosis

**Figure 3:**
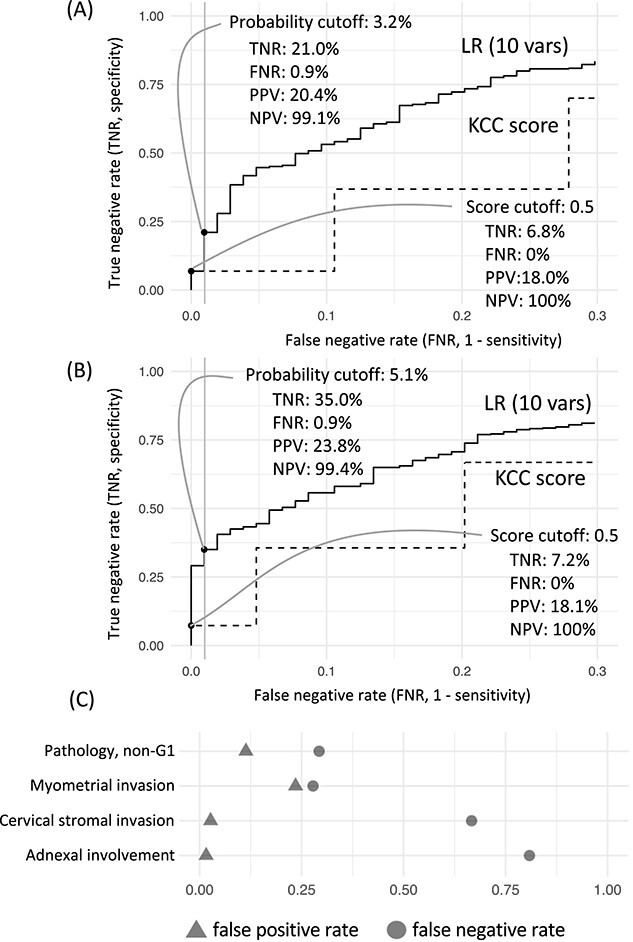
True-negative rates from logistic modelling and ideal preoperative diagnosis. (A) The false-negative (*x-*axis) and true-negative (*y*-axis) rates of the two predictive models, namely our 10-variable LR model (LR 10 vars) and the KCC score. Dots indicate the cutoff to achieve a ~1% false-negative rate, which is indicated by the grey line. (B) The two models are compared as in panel A, except that confirmed postoperative diagnoses were substituted for the preoperative data for pathologic diagnosis and myometrial invasion. (C) Inconsistencies between preoperative and postoperative diagnoses. FNR, false-negative rate; NPV, negative predictive value; PPV, positive predictive value; TNR, true-negative rate.

Because AUC-based assessment of predictive models shows the overall performance with all ranges of cutoffs, the actual usefulness of the predictive model has to be assessed under realistic-use conditions. To assess the improvement in safe omission of lymphadenectomy, or ‘metastasis-free’ diagnosis, the ratio of true negatives under the condition of minimizing false negatives is evaluated. The result showed that, in general, the rate of true negatives was higher for the 10-variable LR model than for the KCC score (Fig. 3A). In particular, when we applied a 1% false-negative rate as the maximum risk—corresponding to accepting only a single false-negative case among 104 cases of LNM in our cohort—the true-negative rate from the 10-variable LR model increased to 21% compared with 6.8% from the KCC score. This 14.2-point increase indicated that the method identified 72 additional LNM-free cases among the 507 LNM-free cases in the cohort.

### Influence of preoperative diagnostic accuracy

Although the regression model increased the true-negative rate, the rate itself remained low (21%), thus raising a question regarding the possibility of further improvement. We therefore examined the accuracy of the four preoperative variables that could be confirmed postoperatively (Fig. 3C). For adnexal involvement and cervical stromal invasion, the rates of false negatives exceeded 60%, confirming the genuine difficulty of using these imaging-based findings. In contrast, the false-negative rates associated with pathologic diagnosis and myometrial invasion were ~25%, thus indicating an opportunity to improve the preoperative prediction of LNM in endometrial cancer by optimizing the diagnostic approach. When we substituted confirmed postoperative findings regarding pathologic diagnosis and myometrial invasion for preoperative data, the true-negative rate of the regression models increased to 35% (Fig. 3B).

### Relationship between estimated LNM probability and KCC score or metastasis type

**Figure 4:**
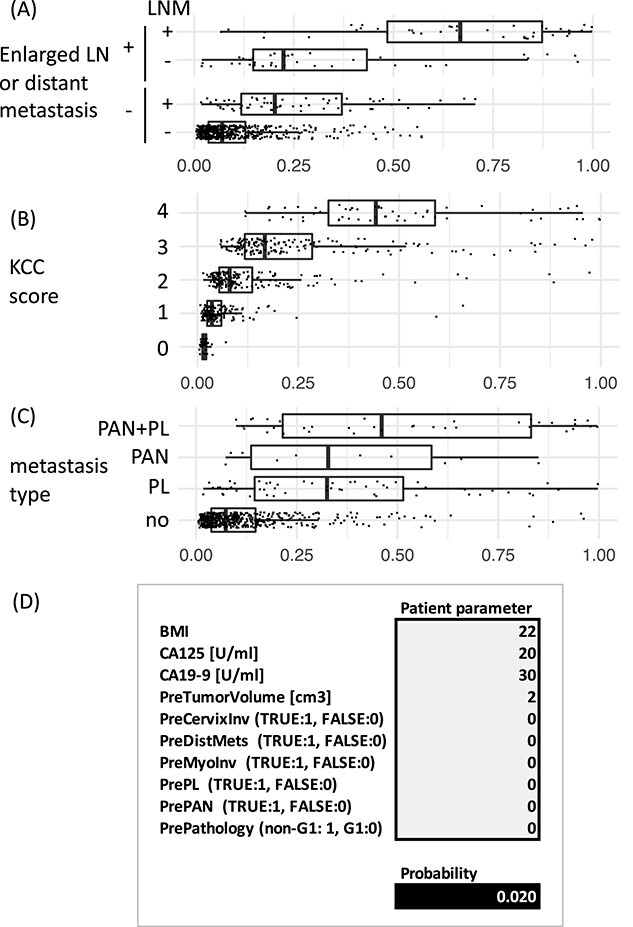
Distribution of predicted probabilities and its spreadsheet-based calculation. Stratified probabilities of our 10-variable logistic model according to (A) very high-risk variables, i.e. enlarged lymph node or distant metastasis (B) KCC score and (C) metastasis type. PAL, para-aortic lymph nodes; PL, pelvic lymph nodes. (D) Example of spreadsheet-based calculation tool for probability prediction of lymph node metastasis by using the logistic model. The spreadsheet itself is available as Supplemental Table S1.

To better understand the probabilities predicted by using our 10-variable LR model, we stratified them according to the presence of the very high-risk variables (i.e. enlarged lymph node and distant metastasis), the KCC score and region of LNM. We found that the metastasis probabilities were lifted in the very high-risk cases, as expected. Even in the remaining cases, their distributions were substantially different among the patients with and without LNM, highlighting the utility of these probabilities in case where the clinical decision is not obvious (Fig. 4A). The probabilities were also reasonably concordant with KCC score (Fig. 4B); however, we found no noteworthy differences between patients with different region of LNM (pelvic, para-aortic or both) (Fig. 4C). Our regression model could be used effectively to predict cases of LNM but not the region affected.

### The calculation tool

Our current 10-variable regression model shows promise for predicting LNM cases in endometrial cancer but needs to be further evaluated before clinical implementation. However, one of the limitations of this model is that it requires a series of in-depth calculations; in contrast, prediction using the KCC scoring system involves merely assessing four factors and counting those that are present. To facilitate further evaluation of our LR model for predicting LNM in endometrial cancer, we prepared a Microsoft Excel spreadsheet to simplify the calculations needed (Fig. 4C and Supplemental Table S1).

## Discussion

Evaluation of LNM is an essential step in guiding therapeutic treatment in endometrial cancer. Because excessive lymphadenectomy markedly reduces the postoperative quality of life, a noninvasive and accurate method for evaluating LNM is needed urgently. We conducted a multi-institutional study to assess the utility of 18 clinical variables in evaluating LNM and found that 10 were significantly associated with LNM. We assessed these 10 variables as prognostic factors according to univariate AUCs and their clinical relevance, and we used them to construct a LR model. We found that the new model (AUC, 0.85; 95% CI, 0.81–0.89; estimated by bootstrapping) significantly outperformed the previous scoring system, namely the KCC score (AUC, 0.74; 95% CI, 0.69–0.79). Our new model not only provided better overall performance (measured as AUC) but also achieved an increased number of correct ‘metastasis-free’ diagnoses; these benefits might increase the opportunities for lymphadenectomy-free treatment, with a false-negative rate limited to <1%. These results underscore the enhanced utility of preoperative clinical variables in LNM evaluation.

Although the improvement of our model over the previous method was significant, we must acknowledge three key limitations in this study. First, the absence of metastasis in the paraaortic lymph node was not pathologically proven in cases where only pelvic lymphadenectomy was performed. We therefore might have underestimated the number of cases of LNM, and our performance assessment might have been somewhat inaccurate. Second, the coefficient of the logistic model was constructed by using this cohort itself. To overcome this limitation, we not only calculated the CIs of the coefficients in the logistic model (Table 2) but also used bootstrapping to assess data-dependent variations in the predictive performance measured according to the AUC (Fig. 2B). Although these combined results support the outstanding predictive performance of the new model, they still reflect a self-contained statistical evaluation, and subsequent validation in an independent cohort is needed. Even though our method requires composite calculations with 10 variables, the calculation tool (Supplemental Table S1) facilitates this process so that individualized risk can be determined for forthcoming patients, thus providing opportunities for follow-up validation studies. Third, the accuracy of imaging-based tests may be limited, likely due to inherent challenges, as highlighted by the absence of a statistically significant association between preoperative identification of adnexal involvement and postoperative diagnosis of LNM. The tests can also vary depending on the instruments, methodologies (MRI and CT) and the institutions where the imaging is conducted. Although our cross-institutional study confirmed the significant contribution of imaging-based tests with the current state, further advancements in methodology and standardization are anticipated to enhance the predictive model for risk assessment.

Noninvasive assessment of LNM using clinical variables has been explored in several studies, including the one responsible for the KCC scoring system (17) and its replication study in southern Iran (20). The Korean Gynecologic Oncology Group defined patients with endometrial cancer at low risk of LNM according to the criteria of myometrial invasion of <50% as assessed by magnetic resonance imaging, no lymphadenopathy or disease progression beyond the uterine corpus and a low CA125 level (35 U/ml); the associated false-negative rate was only 1.3% (negative predictive value, 98.7%; AUC, 0.89; 95% CI, 0.82–0.95) (18,29). By classifying patients into three risk groups (low, intermediate and high) according to the tumour volume index (<36 cm^3^), serum CA125 level (patients younger than 50 years, <70 U/ml; otherwise, <28 U/ml) and a preoperative tumour grading of 1 or 2, Todo et al. achieved a 1.7% false-negative rate (negative predictive value, 98.3%) for the low-risk group (21). By using the criteria of tumour diameter ≤2 cm, low CA125 level (premenopausal, <70 U/ml: postmenopausal, <25 U/ml), computed tomographic scans negative for lymphadenopathy and a preoperative tumour grade of 1 or 2, Matsushita et al. achieved a false-negative rate of 0% (19). Because these previous studies used similar but distinct sets of clinical variables and different thresholds for those variables, we systematically assessed potential clinical variables to construct our predictive method. BMI was unexpectedly found to be associated with LNM, and it is turned out to be consistent with previous studies indicating a link between obesity and the development of endometrial cancer (30) and one between metabolic syndrome risk (MSR) and LNM (31). Employment of multivariate LR led us to only one threshold in the predicted probability, LNM positives or negatives, bypassing multiple and arbitrary thresholds for individual variables. Although our findings cannot be compared directly with those from previous studies, the performance of our model is comparable to those of the earlier methods in terms of AUC values and false-negative rates. In addition, the true-negative rate—an important measure for lymphadenectomy-free treatment—of our new model was substantially better than that for the KCC score, with a comparable false-negative rate.

Additional improvement is needed to achieve fully noninvasive LNM assessment, because the true-negative rate of our method is still unfavourably low. One promising approach for improvement is to use molecular information. The Cancer Genome Atlas (TCGA) defined four subgroups of endometrial cancer through comprehensive genome-wide profiling. It was demonstrated that a compatible classification can be achieved by integrating clinically applicable methods, such as multiple assays of immunohistochemistry and a POLE gene sequencing (32). Furthermore, we previously reported that SAME3D mRNAs and a unique isoform of the TACC2 gene are associated with LNM status (33). Another study proposed a panel of eight gene biomarkers for predicting LNM in patients with early stage endometrial cancer (34). The activation of several pathways involved in biologic processes, such as epithelial–mesenchymal transition, extracellular matrix formation and smooth muscle contraction could be considered (35). Although obtaining molecular signatures at a preoperative stage is currently a challenge, molecular features provide assessment opportunities in addition to those of clinical variables, and their complementary integration might lead to a new scoring system with better predictive performance for LNM in endometrial cancer.

A realistic and minimally invasive approach in LNM evaluation is SLN biopsy. Its overall detection rate is >80% and its specificity is 100% (36). Moreover, the current National Comprehensive Cancer Network (NCCN) guideline version 1.2022 has endorsed SLN mapping with a level 2A evidence category as a technique for the staging of endometrial cancer. Although the utility of SLN biopsy was reported previously in regard to low-risk patients only, its usefulness for high-risk histologic types has been explored recently (37,38). Four prospective cohort trials have shown that SLN biopsy has a high detection rate for pelvic LNM and a high negative predictive value for high-risk and high-grade endometrial cancer (37,39–41). However, regardless of these benefits, SLN biopsy is invasive surgery and requires lymphadenectomy. The probability of surgical complications is never zero (42), and the likelihood of complications increases with wider application (43). One potential use of our statistical model is in prescreening before SLN mapping, so that the use of both non- and minimally invasive treatment modalities might be optimized for minimal risk. We would like to emphasize that the lymph node dissection has proven to be an effective therapeutic method for high-risk patients, but its therapeutic benefit is limited for low-risk patients (14,15). Meanwhile, it is essential to conduct risk assessment for all patients regardless of their risk groups. Therefore, if the preoperative risk assessment suggests the omission of lymphadenectomy for low-risk patients, even if it is an underestimation, it does not imply inappropriate treatment. Diagnostic tests and therapeutic treatments will have to be designed separately based on the available methods and evidences of effectiveness.

In conclusion, we developed a preoperative scoring system that is based solely on clinical variables to predict LNM in endometrial cancer. Our new method might be used for noninvasive LNM assessment, during prescreening for SLN mapping, and in guiding lymphadenectomy. Although further assessment is required, the predictive performance of our method appears to be substantially improved over those of previous scoring systems. In addition, we created a spreadsheet-based tool to simplify the process of statistical risk score calculation associated with our method. Regardless of additional challenges, including the incorporation of biomarkers into our scoring system, our results advance non- or minimally invasive LNM assessment one step further.

## Supplementary Material

06_Ueno_et_al_EMca_LMNpred_tables_suppl_230104_hyad135

06_Ueno_et_al_EMca_LNMpred_tables_suppl_231001_hyad135

## Data Availability

The data generated in this study are available upon reasonable request from the corresponding author.
